# Two-Level Finite Element Iterative Algorithm Based on Stabilized Method for the Stationary Incompressible Magnetohydrodynamics

**DOI:** 10.3390/e24101426

**Published:** 2022-10-07

**Authors:** Qili Tang, Min Hou, Yajie Xiao, Lina Yin

**Affiliations:** Hunan Key Laboratory for Computation and Simulation in Science and Engineering, Key Laboratory of Intelligent Computing & Information Processing of Ministry of Education, School of Mathematics and Computational Science, Xiangtan University, Xiangtan 411105, China

**Keywords:** finite element method, two-level method, stabilized method, Oseen iteration, stationary incompressible MHD, 35Q30, 65M60, 65N30, 76D05

## Abstract

In this paper, based on the stabilization technique, the Oseen iterative method and the two-level finite element algorithm are combined to numerically solve the stationary incompressible magnetohydrodynamic (MHD) equations. For the low regularity of the magnetic field, when dealing with the magnetic field sub-problem, the Lagrange multiplier technique is used. The stabilized method is applied to approximate the flow field sub-problem to circumvent the inf-sup condition restrictions. One- and two-level stabilized finite element algorithms are presented, and their stability and convergence analysis is given. The two-level method uses the Oseen iteration to solve the nonlinear MHD equations on a coarse grid of size *H*, and then employs the linearized correction on a fine grid with grid size *h*. The error analysis shows that when the grid sizes satisfy $h=O(H^{2})$, the two-level stabilization method has the same convergence order as the one-level one. However, the former saves more computational cost than the latter one. Finally, through some numerical experiments, it has been verified that our proposed method is effective. The two-level stabilized method takes less than half the time of the one-level one when using the second class Nédélec element to approximate magnetic field, and even takes almost a third of the computing time of the one-level one when adopting the first class Nédélec element.

## 1. Introduction

Consider the following stationary incompressible MHD

(1) $$\begin{array}{cc} -R_{e}^{-1}\Delta u+\left(u\cdot ▽\right)u+\nabla p-S_{c}\mathrm{curl}b\times b & =f,\;\;\mathrm{in}\;\;\Omega ,\\ R_{m}^{-1}S_{c}\mathrm{curl}\left(\mathrm{curl}b\right)-S_{c}\mathrm{curl}\left(u\times b\right)-\nabla r & =g,\;\;\mathrm{in}\;\;\Omega ,\\ \mathrm{div}u=0,\;\;\mathrm{div}b & =0,\;\;\mathrm{in}\;\;\Omega ,\\ u=0,\;\;b\cdot n=0,\;\;n\times \mathrm{curl}b=0,\;\;r & =0,\;\;\mathrm{on}\;\;\partial \Omega , \end{array}$$

where $\Omega \in R^{d}$ (d=2,3) is a bounded Lipschitz domain. $R_{e}$ and $R_{m}$ are the hydrodynamic and magnetic Reynolds numbers, respectively. $S_{c}$ is the coupling number, and f and g are source terms with ∇·g=0. n is the unit outward normal vector on ∂Ω.

Incompressible MHD describes the dynamics of a viscous, incompressible, electrically conducting fluid under an external magnetic field. The MHD (1) is a coupled multi-physical system of the classical Navier–Stokes equations and Maxwell’s equations. MHD modelling has a number of applications in physics and engineering technology, such as radio wave propagation in ionosphere in geophysics, MHD engine, control of MHD boundary layer and liquid-metal MHD electricity generation (see [1]). Since MHD equations are strongly nonlinear and have many physical quantities, it is needed to find effective numerical methods to solve them.

For the MHD modelling (1) without the Lagrange multiplier *r* term, the early study of the exact penalty regularization finite element method on a convex domain is carried out in [2]. Based on this format, the nonconforming mixed finite element methods [3], the Stokes, Newton and Oseen finite element iterative methods [4,5], the penalty based finite element iterative methods [6], and the generalized Arrow–Hurwicz iterative methods [7] are investigated. In view of multi-physical coupling and nonlinearity of system (1), two-level method and finite element iterative algorithms are combined by [8,9,10,11,12] to reduce the computing cost, and local and parallel finite element algorithms based on some iterations are proposed in [13,14,15,16]. On the other hand, a number of effective solvers based on the finite element methods are presented in [17,18,19]. To keep the physical property of the Gauss law of the magnetic field, the constrained transport divergence-free finite element method is designed in [20]. The coupled Stokes, Newton and Oseen-type iteration methods are studied and discussed for the (1) in [21] on a general Lipschitz domain. For the nonsmooth computational domain, the magnetic field belongs to a lower regularity space than $H^{1}\left(\Omega \right)$, and the discrete finite element scheme with the Lagrange multiplier of (1) becomes a double-saddle points problem.

For the mixed finite element method, the component approximations must preserve the compatibility and satisfy the so-called inf-sup condition. It is well known that the lowest equal-order finite element pairs in engineering preferred do not satisfy the inf-sup condition. Numerical experiments show that the break of the inf-sup condition often leads to unphysical pressure oscillations. To avoid the instability problem and use the lowest equal-order elements, the popular stabilized methods based on local Gauss integrations are proposed and studied, for example, for the Stokes problem [22,23], the coupled Stokes–Darcy problem [24], the Stokes eigenvalue system [25], the Navier–Stokes equations [26,27,28,29] and the natural convection problem [30]. However, the stabilized finite element algorithm for MHD with respect to the Lagrange multiplier has not been reported.

In this paper, a two-level finite element iterative algorithm based on the stabilized method is proposed to numerically solve the stationary incompressible MHD equations. Compared to the existing literature, the stabilized scheme with the Lagrange multiplier proposed here have two main benefits. One is that the lowest equal-order finite element pairs can be used to approximate hydrodynamic subproblem, and the other is that our scheme preserve the physical property of Gauss law weakly for magnetic subproblem by adding the Lagrange multiplier. In the next section, the stabilized finite element discretization based on local Gauss integrations is designed and analyzed. To deal with the nonlinear term, the stabilized finite element method based on Oseen iteration is studied. The two-level stabilized finite element algorithm and its convergence are given in Section 3. In the last section, some numerical experiments are tested to support the theoretical analysis of our proposed method.

## 2. Stabilized Finite Element Discretization Based on Local Gauss Integrations

We will introduce some Sobolev spaces, and the norms of the product spaces:

$$\begin{array}{c} H^{1}\left(\Omega \right)=H^{1}{\left(\Omega \right)}^{d},\;X:=H_{0}^{1}\left(\Omega \right)=\left\{u\in H^{1}\left(\Omega \right):u{|}_{\partial \Omega }=0\right\},\\ H\left(\mathrm{div};\Omega \right)=\{u\in L^{2}{\left(\Omega \right)}^{d}:\mathrm{div}u\in L^{2}\left(\Omega \right)\},\\ W:=H_{0}\left(\mathrm{curl};\Omega \right)=\left\{b\in H\left(\mathrm{curl};\Omega \right):b\times n{|}_{\partial \Omega }=0\right\},\\ Q:=L_{0}^{2}\left(\Omega \right)=\left\{q\in L^{2}\left(\Omega \right):\int_{\Omega }qdx=0\right\},S:=H_{0}^{1}\left(\Omega \right),\\ {\left||\left(u,b\right)|\right|}_{1}={\left({\left||\nabla u|\right|}_{0}^{2}+S_{c}{\left||\mathrm{curl}b|\right|}_{0}^{2}\right)}^{\frac{1}{2}},\;||\left(p,r\right)||={\left({\left||p|\right|}_{0}^{2}+{\left||\nabla r|\right|}_{0}^{2}\right)}^{\frac{1}{2}},\\ {\left||\left(u,b\right)|\right|}_{0}={\left({\left||u|\right|}_{0}^{2}+S_{c}{\left||b|\right|}_{0}^{2}\right)}^{\frac{1}{2}}{,||f||}_{-1}=\underset{v\in H_{0}^{1}\left(\Omega \right)}{\sup}\frac{\langle f,v\rangle }{{\left||\nabla v|\right|}_{0}},\\ {\left||F|\right|}_{*}={\left({\left||f|\right|}_{-1}^{2}+S_{c}^{-1}{\left||g|\right|}_{0}^{2}\right)}^{\frac{1}{2}}{,\;||F||}_{0}={\left({\left||f|\right|}_{0}^{2}+S_{c}^{-1}{\left||g|\right|}_{0}^{2}\right)}^{\frac{1}{2}}. \end{array}$$

For all w,u,v∈X,b,c,d∈W,q∈Q,s∈S, let

$$\begin{array}{c} a_{s}\left(u,v\right)=R_{e}^{-1}\left(\nabla u,\nabla v\right),a_{m}\left(b,c\right)=R_{m}^{-1}S_{c}\left(\mathrm{curl}b,\mathrm{curl}c\right),\\ c_{0}\left(w,u,v\right)=\left(w\cdot \nabla u,v\right)=\frac{1}{2}\left(w\cdot \nabla u,v\right)-\frac{1}{2}\left(w\cdot \nabla v,u\right),\\ c_{1}\left(d,v,b\right)=S_{c}\left(\mathrm{curl}b\times d,v\right),\\ b_{s}\left(q,v\right)=\left(q,\nabla \cdot v\right),b_{m}\left(s,c\right)=\left(\nabla s,c\right),\\ A\left(u,b;v,c\right)=a_{s}\left(u,v\right)+a_{m}\left(b,c\right),\\ C\left(w,d;u,b;v,c\right)=c_{0}\left(w,u,v\right)-c_{1}\left(d,v,b\right)+c_{1}\left(d,u,c\right),\\ B\left(q,s;v,c\right)=b_{s}\left(q,v\right)+b_{m}\left(s,c\right),\\ \langle F,(v,c)\rangle =\langle f,v\rangle +(g,c). \end{array}$$

By the Lagrange multiplier technique, the variational form of system (1) is [31]: Find (u,b,p,r)∈X×W×Q×S, for all (v,c,q,s)∈X×W×Q×S such that

(2) $$\begin{array}{cc} \; & a_{s}\left(u,v\right)-b_{s}\left(p,v\right)+b_{s}\left(q,u\right)+c_{0}\left(u,u,v\right)-c_{1}\left(b,v,b\right)=\langle f,v\rangle , \end{array}$$

(3) $$\begin{array}{c} a_{m}\left(b,c\right)-b_{m}\left(r,c\right)+b_{m}\left(s,b\right)+c_{1}\left(b,u,c\right)=\left(g,c\right). \end{array}$$

The compact form of (2) and (3) is read as

(4) $$\begin{array}{cc} A(u,b;v,c)+C(u,b;u,b;v,c)-B(p,r;v,c)+B(q,s;u,b) & =\langle F,(v,c)\rangle . \end{array}$$

The properties of the bilinear and trilinear forms from [32,33,34] are useful for our analysis. For all u,v∈X,b,c∈W,q∈Q,r∈S, there have

(5) $$\begin{array}{c} \left|A\left(u,b;v,c\right)\right|\leq \bar{v}{\left||\left(u,b\right)|\right|}_{1}{\left||\left(v,c\right)|\right|}_{1},\bar{v}=\max\left\{R_{e}^{-1},R_{m}^{-1}\right\}, \end{array}$$

(6) $$\begin{array}{c} A\left(u,b;u,b\right)\geq \underline{v}{\left||\left(u,b\right)|\right|}_{1}^{2},\underline{v}=\min\left\{R_{e}^{-1},R_{m}^{-1}\lambda_{0}\right\}, \end{array}$$

(7) $$\begin{array}{c} B\left(q,s;v,c\right)\leq \sqrt{d}{\left||\left(q,s\right)||||\left(v,c\right)|\right|}_{1}, \end{array}$$

(8) $$\begin{array}{c} C(w,d;u,b;u,b)=0, \end{array}$$

(9) $$\begin{array}{c} \left|C\left(w,d;u,b;v,c\right)\right|\leq \hat{N}{\left||\left(w,d\right)|\right|}_{1}{\left||\left(u,b\right)|\right|}_{1}{\left||\left(v,c\right)|\right|}_{1}, \end{array}$$

where $\hat{N}$ and $\lambda_{0}$ are positive constants that depend only on Ω. In the next content, we use *C* to represent a general positive constant independent of mesh sizes *H* and *h*.

*H* and *h* (h≪H) are now two real positive parameters that tend to 0. $T_{H}$ is a uniformly regular partition of Ω into triangular (d=2) or tetrahedral (d=3) element *K* with diameters bounded by *H*, and $T_{h}$ is the fine mesh generated by a mesh refinement process to $T_{H}$. Let $T_{\mu }\left(\mu =H,h\right)$ is a partition. $P_{k}\left(K\right)$ is the space of polynomials of degree *k* (positive integers) over *K*. $P_{1}$ element is utilized to approximate the velocity field, pressure and Lagrange multiplier, and two kinds of lowest order Nédélec elements are applied to approximate the magnetic field. The subspaces of X,W,Q,S are

$$\begin{array}{cc} \; & X_{\mu }:=\left\{u_{\mu }\in H_{0}^{1}\left(\Omega \right):u_{\mu }{|}_{K}\in P_{1}{\left(K\right)}^{d},\forall K\in T_{\mu }\right\},\\ \; & W_{\mu }:=\left\{b_{\mu }\in H_{0}\left(\mathrm{curl};\Omega \right):b_{\mu }{|}_{K}\in N_{1}^{\left(l\right)}\left(K\right),\forall K\in T_{\mu }\right\},l=1,2,\\ \; & Q_{\mu }:=\left\{q_{\mu }\in L_{0}^{2}\left(\Omega \right):q_{\mu }{|}_{K}\in P_{1}\left(K\right),\forall K\in T_{\mu }\right\},\\ \; & S_{\mu }:=\left\{r_{\mu }\in H_{0}^{1}\left(\Omega \right):r_{\mu }{|}_{K}\in P_{1}\left(K\right),\forall K\in T_{\mu }\right\},\\ \; & V_{\mu }:=\left\{u_{\mu }\in X_{\mu }:b_{s}\left(q_{\mu },u_{\mu }\right)=0,\forall q_{\mu }\in Q_{\mu }\right\},\\ \; & C_{\mu }:=\left\{b_{\mu }\in W_{\mu }:b_{m}\left(s_{\mu },b_{\mu }\right)=0,\forall s_{\mu }\in S_{\mu }\right\}. \end{array}$$

Here, Nédélec elements of the first family and the second one are as follows [35]

$$\begin{array}{cc} \; & N_{k}^{\left(1\right)}\left(K\right)={\left[P_{k-1}\left(K\right)\right]}^{d}⊕D_{k}\left(K\right),D_{k}\left(K\right)=\left.\left\{p\in {\left[{\tilde{P}}_{k}\left(K\right)\right]}^{d}\right|\;p\left(x\right)\cdot x=0,\;\mathrm{in}\;K\right\},\\ \; & N_{k}^{\left(2\right)}\left(K\right)={\left[P_{k}\left(K\right)\right]}^{d}, \end{array}$$

where ${\left[{\tilde{P}}_{k}\left(K\right)\right]}^{d}$ is the homogeneous polynomials of degree *k*.

$W_{\mu }$ and $S_{\mu }$ satisfy the discrete inf-sup condition [31]

(10) $$\begin{array}{c} \underset{c_{\mu }\in W_{\mu }}{\sup}\frac{b_{m}\left(s_{\mu },c_{\mu }\right)}{∥\mathrm{curl}c_{\mu }{∥}_{0}}\geq \hat{\beta }{∥\nabla s_{\mu }∥}_{0},\forall s_{\mu }\in S_{\mu }, \end{array}$$

where the constant $\hat{\beta }>0$ is independent of μ.

Denote $P_{\mu }$ and $R_{0\mu }$ by the $L^{2}$-orthogonal projectors

$$P_{\mu }:L^{2}\left(\Omega \right)\to V_{\mu },\;R_{0\mu }:L^{2}\left(\Omega \right)\to C_{\mu }.$$

Define the discrete Stokes operator by $A_{1\mu }=-P_{\mu }\Delta_{\mu }$, in which $\Delta_{\mu }$ is defined by (see [32,33])

$$-\left(\Delta_{\mu }u_{\mu },v\right)=\left(\nabla u_{\mu },\nabla v\right),\;\forall u_{\mu },\;v\in X_{\mu },$$

and the discrete norm $∥u_{\mu }{∥}_{k,\mu }={∥A_{1\mu }^{\frac{k}{2}}u_{\mu }∥}_{0}$ of the k∈R order, where

$$∥u_{\mu }{∥}_{1,\mu }=∥\nabla u_{\mu }{∥}_{0},\;∥u_{\mu }{∥}_{2,\mu }={∥A_{1\mu }u_{\mu }∥}_{0},\;\forall u_{\mu }\in X_{\mu }.$$

Meanwhile, $A_{2\mu }b_{\mu }=R_{0\mu }\left(\nabla_{\mu }\times \nabla \times b_{\mu }+b_{\mu }\right)$ is defined as [33]:

$$\left(\nabla_{\mu }\times \nabla \times b_{\mu },c\right)=\left(\nabla \times b_{\mu },\nabla \times c\right),\;\forall c\in W_{\mu }.$$

It is necessary to introduce some discrete estimates [33,34]

(11) $$\begin{array}{c} ∥\nabla v_{\mu }{∥}_{L^{3}}+∥v_{\mu }{∥}_{L^{\infty }}\leq ∥\nabla v_{\mu }{∥}_{0}^{\frac{1}{2}}{∥A_{1\mu }v_{\mu }∥}_{0}^{\frac{1}{2}},\\ ∥\nabla v_{\mu }{∥}_{L^{6}}\leq C{∥A_{1\mu }v_{\mu }∥}_{0},\;\forall v_{\mu }\in X_{\mu }. \end{array}$$

The trilinear form C(·,·,·) has the properties [34]: for all $w_{\mu },u_{\mu },v_{\mu }\in X_{\mu },d_{\mu },b_{\mu },c_{\mu }\in \;W_{\mu }$,

(12) $$\begin{array}{c} |C\left(w_{\mu },d_{\mu };u_{\mu },b_{\mu };v_{\mu },c_{\mu }\right)|+|C\left(u_{\mu },b_{\mu };w_{\mu },d_{\mu };v_{\mu },c_{\mu }\right)|\\ \;\leq \hat{N}∥\left(A_{1\mu }w_{\mu },A_{2\mu }d_{\mu }\right){∥}_{0}∥\left(u_{\mu },b_{\mu }\right){∥}_{0}{∥\left(v_{\mu },c_{\mu }\right)∥}_{1}. \end{array}$$

It is apparent that the discrete inf-sup condition is not valid to the subspace $X_{\mu }$ and $Q_{\mu }$. To meet the needs of this property, as in [22,26], a mixed stability term with the universal bilinear form is added:

(13) $$\begin{array}{c} D_{\mu }\left(u_{\mu },p_{\mu };v_{\mu },q_{\mu }\right)=a_{s}\left(u_{\mu },v_{\mu }\right)-b_{s}\left(p_{\mu },v_{\mu }\right)+b_{s}\left(q_{\mu },u_{\mu }\right)+G\left(p_{\mu },q_{\mu }\right), \end{array}$$

where

$$\begin{array}{c} G\left(p_{\mu },q_{\mu }\right)=\sum_{K\in T_{\mu }}\left(\int_{K,k}p_{\mu }q_{\mu }d\xi -\int_{K,1}p_{\mu }q_{\mu }d\xi \right),\;k⩾2, \end{array}$$

for all $p_{\mu },q_{\mu }\in Q_{\mu }$, $\int_{K,i}p_{\mu }q_{\mu }d\xi$ means that makes use of an *i*-order (i=1,2) local Gauss integral to calculate it over the element K.

Let $\Pi_{\mu }:L^{2}\left(\Omega \right)\to P_{0}$ be a $L^{2}$-projection with the properties as follows [22,36]:

(14) $$\begin{array}{c} \left(p,q_{\mu }\right)=\left(\Pi_{\mu }p,q_{\mu }\right),\forall p\in L^{2}\left(\Omega \right),q_{\mu }\in Q_{\mu },\\ ∥\Pi_{\mu }{p∥}_{0}\leq C{∥p∥}_{0},\forall p\in L^{2}\left(\Omega \right),\\ ∥p-\Pi_{\mu }{p∥}_{0}\leq C\mu^{\min\{1,\gamma \}}{∥p∥}_{\gamma },\forall p\in H^{1}\left(\Omega \right)\cap H^{\gamma }\left(\Omega \right). \end{array}$$

As a consequence, the local Gauss integral can be restated as:

(15) $$\begin{array}{c} G\left(p_{\mu },q\right)=\left(p_{\mu }-\Pi_{\mu }p_{\mu },q-\Pi_{\mu }q\right),\;\forall p_{\mu },q\in Q_{\mu }. \end{array}$$

$D_{\mu }\left(\cdot ,\cdot ;\cdot ,\cdot \right)$ satisfies the following important properties (see [22,26]): For all $\left(u_{\mu },p_{\mu }\right),\left(v_{\mu },q_{\mu }\right)\in X_{\mu }\times Q_{\mu }$,

(16) $$\begin{array}{c} \underset{\left(v,q\right)\in \left(X_{\mu },Q_{\mu }\right)}{\sup}\frac{|D_{\mu }\left(u_{\mu },p_{\mu };v,q\right)|}{{∥\nabla v∥}_{0}+{∥q∥}_{0}}\geq \hat{\beta }(R_{e}^{-1}∥\nabla u_{\mu }{∥}_{0}+∥p_{\mu }{∥}_{0}). \end{array}$$

The stabilized discrete scheme reads: Find $\left(u_{\mu },b_{\mu },p_{\mu },r_{\mu }\right)\in X_{\mu }\times W_{\mu }\times Q_{\mu }\times S_{\mu },$ for all $\left(v,c,q,s\right)\in X_{\mu }\times W_{\mu }\times Q_{\mu }\times S_{\mu }$, such that

(17) $$\begin{array}{c} \begin{array}{c} a_{s}\left(u_{\mu },v\right)+c_{0}\left(u_{\mu },u_{\mu },v\right)-c_{1}\left(b_{\mu },v,b_{\mu }\right)-b_{s}\left(p_{\mu },v\right)+b_{s}\left(q,u_{\mu }\right)\\ \;+G\left(p_{\mu },q\right)+\frac{\sigma \mu }{R_{e}^{-1}}a_{s}\left(u_{\mu },v\right)=\langle f,v\rangle ,\\ a_{m}\left(b_{\mu },c\right)+c_{1}\left(b_{\mu },u_{\mu },c\right)-b_{m}\left(r_{\mu },c\right)+b_{m}\left(s,b_{\mu }\right)=\left(g,c\right), \end{array} \end{array}$$

where σ>0 is an artificial viscosity parameter, (17) can be rewritten as:

(18) $$\begin{array}{c} A\left(u_{\mu },b_{\mu },\;v,c\right)+C\left(u_{\mu },b_{\mu };u_{\mu },b_{\mu };v,c\right)+\frac{\sigma \mu }{R_{e}^{-1}}a_{s}\left(u_{\mu },v\right)+G\left(p_{\mu },q\right)\\ \;-B\left(p_{\mu },r_{\mu };v,c\right)+B\left(q,s;u_{\mu },b_{\mu }\right)=\langle F,\left(v,c\right)\rangle . \end{array}$$

Let

$$A_{\mu }\left(u_{\mu },b_{\mu };v,c\right)=A\left(u_{\mu },b_{\mu };v,c\right)+\frac{\sigma \mu }{R_{e}^{-1}}a_{s}\left(u_{\mu },v\right).$$

Then the bilinear form $A_{\mu }$ satisfies the following coercive and continuous properties:

(19) $$\begin{array}{c} |A_{\mu }\left(u_{\mu },b_{\mu };v,c\right)|\leq C_{max}∥\left(u_{\mu },b_{\mu }\right){∥}_{1}{∥\left(v,c\right)∥}_{1}, \end{array}$$

(20) $$\begin{array}{c} A_{\mu }\left(u_{\mu },b_{\mu };u_{\mu },b_{\mu }\right)\geq C_{min}{∥\left(u_{\mu },b_{\mu }\right)∥}_{1}^{2}, \end{array}$$

where

$$C_{max}=\left\{R_{e}^{-1}+\sigma \mu ,R_{m}^{-1}\right\},C_{min}=\left\{R_{e}^{-1}+\sigma \mu ,\;R_{m}^{-1}\lambda_{0}\right\}.$$

Rewrite (18) as

(21) $$\begin{array}{c} A_{\mu }\left(u_{\mu },b_{\mu },v,c\right)+C\left(u_{\mu },b_{\mu };u_{\mu },b_{\mu };v,c\right)+G\left(p_{\mu },q\right)\\ \;-B\left(p_{\mu },r_{\mu };v,c\right)+B\left(q,s;u_{\mu },b_{\mu }\right)=\langle F,\left(v,c\right)\rangle . \end{array}$$

In order to derive error estimates, we introduce two projections. The Stokes projection is defined as follows [26,36] : Find $R\left(u,p\right)\in X_{\mu },Q\left(u,p\right)\in Q_{\mu }$ such that

(22) $$\begin{array}{c} a_{s}\left(u-R\left(u,p\right),v\right)-b_{s}\left(p-Q\left(u,p\right),v\right)+b_{s}\left(q,u-R\left(u,p\right)\right)=0, \end{array}$$

for all $\left(v,q\right)\in X_{\mu }\times Q_{\mu }$. If $u\in H^{1+\gamma }\left(\Omega \right),p\in H^{\gamma }\left(\Omega \right),\gamma >\frac{1}{2}$, there holds [26,36]

(23) $$\begin{array}{cc} & {∥u-R\left(u,p\right)∥}_{0}+{\mu (∥\nabla \left(u-R\left(u,\;p\right)\right)∥}_{0}{+∥p-Q\left(u,p\right)∥}_{0})\\ & \leq C\mu^{\min\{2,\gamma +1\}}{(∥u∥}_{1+\gamma }+{∥p∥}_{\gamma }). \end{array}$$

The Maxwell’s projection is defined by [37]: Assume that $b\in H^{\tau }\left(\Omega \right),\mathrm{curl}b\in H^{\tau }\left(\Omega \right),\tau >\frac{1}{2}$, find $\Lambda b\in W_{\mu },\Lambda r\in S_{\mu }$ such that

(24) $$\begin{array}{c} a_{m}\left(b-\Lambda b,c\right)-b_{m}\left(r-\Lambda r,c\right)+b_{m}\left(s,\;b-\Lambda b\right)=0,\;\forall b\in W,r\in S. \end{array}$$

By the property of Λ, it can be shown that

(25) $$\begin{array}{c} {∥\mathrm{curl}b-\mathrm{curl}\Lambda b∥}_{0}{+∥b-\Lambda b∥}_{0}+{∥\nabla \left(r-\Lambda r\right)∥}_{0}\\ \;\leq C\mu^{\min\{1,\tau \}}\left({∥b∥}_{\tau }+{∥\mathrm{curl}b∥}_{\tau }+{∥r∥}_{\tau +1}\right). \end{array}$$

Now, we will give the stability and error estimate for the problem (21).

**Lemma** **1.** Suppose the condition $\sigma_{1}:=\frac{\hat{N}{∥F∥}_{*}}{{\left(C_{min}\right)}^{2}}<1$ holds, the solution of the problem (21) satisfies

(26) $$\begin{array}{c} C_{min}∥\left(u_{\mu },\;b_{\mu }\right){∥}_{1}\leq {∥F∥}_{*},\; \end{array}$$

(27) $$\begin{array}{c} C_{min}\left(1-\sigma_{1}\right)∥\left(A_{1\mu }u_{\mu },A_{2\mu }b_{\mu }\right){∥}_{0}\leq {∥F∥}_{0}. \end{array}$$

**Proof.**  For (21), taking $\left(v,b,q,s\right)=\left(u_{\mu },b_{\mu },p_{\mu },r_{\mu }\right)\in X_{\mu }\times W_{\mu }\times Q_{\mu }\times S_{\mu }$, then by (8), we have

$$\begin{array}{c} A_{\mu }\left(u_{\mu },b_{\mu },u_{\mu },b_{\mu }\right)+G\left(p_{\mu },p_{\mu }\right)=\langle F,\left(u_{\mu },b_{\mu }\right)\rangle . \end{array}$$

Using (20) and (15), we can easily receive (26).

Replacing $\left(v,c\right)=\left(A_{1\mu }u_{\mu },A_{2\mu }b_{\mu }\right),q=0,s=0$ in (21), and applying (12) to have

$$\begin{array}{c} \begin{array}{cc} C_{min}{∥\left(A_{1\mu }u_{\mu },\;A_{2\mu }b_{\mu }\right)∥}_{0}\; & \leq {∥F∥}_{0}+\hat{N}∥\left(A_{1\mu }u_{\mu },\;A_{2\mu }b_{\mu }\right){∥}_{0}{∥\left(u_{\mu },\;b_{\mu }\right)∥}_{1}\\ \; & \leq {∥F∥}_{0}+\hat{N}{∥\left(A_{1\mu }u_{\mu },A_{2\mu }b_{\mu }\right)∥}_{0}\frac{{∥F∥}_{*}}{C_{min}}. \end{array} \end{array}$$

Furthermore, we arrive at (27).    □

**Theorem** **1.** *Let (u,b,p,r) be the solution of the problem (4) satisfying $u\in H^{1+\gamma }\left(\Omega \right),p\in H^{\gamma }\left(\Omega \right),b\in H^{\tau }\left(\Omega \right),\mathrm{curl}b\in H^{\tau }\left(\Omega \right),r\in H^{1+\tau }\left(\Omega \right)$, $\gamma ,\tau >\frac{1}{2}.$ Then, the error estimate $\left(u-u_{\mu },b-b_{\mu }\right)$ and $\left(p-p_{\mu },r-r_{\mu }\right)$ of the solution (18) satisfying the upper bound*

(28) $$\begin{array}{c} \begin{array}{c} ∥\left(u-u_{\mu },b-b_{\mu }\right){∥}_{1}\\ \;\leq C\mu^{\min\{1,\gamma ,\tau \}}{(∥u∥}_{1+\gamma }+{∥b∥}_{\tau }+{∥\mathrm{curl}b∥}_{\tau }+{∥p∥}_{\gamma }+{∥r∥}_{1+\tau })\\ \;\;+{C\sigma \mu ∥F∥}_{*}. \end{array} \end{array}$$

(29) $$\begin{array}{c} \begin{array}{c} ∥(p-p_{\mu },r-r_{\mu })∥\\ \;\leq C\mu^{\min\{1,\gamma ,\tau \}}{(∥u∥}_{1+\gamma }+{∥b∥}_{\tau }+{∥\mathrm{curl}b∥}_{\tau }+{∥p∥}_{\gamma }+{∥r∥}_{1+\tau })\\ \;\;+{C\sigma \mu ∥F∥}_{*}. \end{array} \end{array}$$

(30) $$\begin{array}{c} ∥\left(u-u_{\mu },b-b_{\mu }\right){∥}_{0}\\ \;\leq C\mu^{\min\{2,\gamma +1,\tau +1\}}{(∥u∥}_{1+\gamma }+{∥b∥}_{\tau }+{∥\mathrm{curl}b∥}_{\tau }+{∥p∥}_{\gamma }+{∥r∥}_{1+\tau })+C\mu^{2}. \end{array}$$

The proof of Theorem 1 is shown in the section of Appendix A.

In the following, the Oseen iteration is used to linearize the stabilized finite element discrete form (17). The stability and convergence is proven. The stabilized finite element algorithm based on Oseen iteration is stated as follows: Given $\left(u_{\mu }^{n-1},b_{\mu }^{n-1},p_{\mu }^{n-1},r_{\mu }^{n-1}\right)$, find $\left(u_{\mu }^{n},b_{\mu }^{n},p_{\mu }^{n},r_{\mu }^{n}\right)\in X_{\mu }\times W_{\mu }\times Q_{\mu }\times S_{\mu }$ such that

(31) $$\begin{array}{c} a_{s}\left(u_{\mu }^{n},v\right)+c_{0}\left(u_{\mu }^{n-1},\;u_{\mu }^{n},v\right)-c_{1}\left(b_{\mu }^{n-1},v,b_{\mu }^{n}\right)-b_{s}\left(p_{\mu }^{n},v\right)+b_{s}\left(q,u_{\mu }^{n}\right)\\ \;+G\left(p_{\mu }^{n},q\right)+\frac{\sigma \mu }{R_{e}^{-1}}a_{s}\left(u_{\mu }^{n},v\right)=\langle f,v\rangle , \end{array}$$

(32) $$\begin{array}{c} a_{m}\left(b_{\mu }^{n},c\right)+c_{1}\left(b_{\mu }^{n-1},u_{\mu }^{n},\;c\right)-b_{m}\left(r_{\mu }^{n},c\right)+b_{m}\left(s,b_{\mu }^{n}\right)=\left(g,c\right). \end{array}$$

Here, the initial value $\left(u_{\mu }^{0},b_{\mu }^{0},p_{\mu }^{0},r_{\mu }^{0}\right)\in X_{\mu }\times W_{\mu }\times Q_{\mu }\times S_{\mu }$ is given by

(33) $$\begin{array}{c} a_{s}\left(u_{\mu }^{0},v\right)-b_{s}\left(p_{\mu }^{0},v\right)+b_{s}\left(q,u_{\mu }^{0}\right)+G\left(p_{\mu }^{0},q\right)+\frac{\sigma \mu }{R_{e}^{-1}}a_{s}\left(u_{\mu }^{0},v\right)=\langle f,v\rangle , \end{array}$$

(34) $$\begin{array}{c} a_{m}\left(b_{\mu }^{0},c\right)-b_{m}\left(r_{\mu }^{0},c\right)+b_{m}\left(s,b_{\mu }^{0}\right)=\left(g,c\right). \end{array}$$

Rewrite (31) and (32) in compact form as

(35) $$\begin{array}{c} A_{\mu }\left(u_{\mu }^{n},b_{\mu }^{n},v,c\right)+C\left(u_{\mu }^{n-1},b_{\mu }^{n-1};u_{\mu }^{n},b_{\mu }^{n};v,c\right)+G\left(p_{\mu }^{n},q\right)-B\left(p_{\mu }^{n},r_{\mu }^{n};v,c\right)\\ \;+B\left(q,s;u_{\mu }^{n},b_{\mu }^{n}\right)=\langle F,\left(v,c\right)\rangle , \end{array}$$

for n=1,2,⋯, for all $\left(v,c,q,s\right)\in X_{\mu }\times W_{\mu }\times Q_{\mu }\times S_{\mu }$.

**Lemma** **2.** *If the condition $0<\sigma_{1}<1$ holds, for all m≥0, then the solution $\left(u_{\mu }^{m},b_{\mu }^{m},p_{\mu }^{m},r_{\mu }^{m}\right)$ of (35) satisfies*

(36) $$\begin{array}{c} C_{min}∥\left(u_{\mu }^{m},b_{\mu }^{m}\right){∥}_{1}+∥\left(p_{\mu }^{m},r_{\mu }^{m}\right){∥\leq C∥F∥}_{*}, \end{array}$$

(37) $$\begin{array}{c} ∥\left(A_{1\mu }u_{\mu }^{m},A_{2\mu }b_{\mu }^{m}\right){∥}_{0}\leq C{∥F∥}_{0}. \end{array}$$

**Proof.**  For m=0, taking $\left(v,b,q,s\right)=\left(u_{\mu }^{0},b_{\mu }^{0},p_{\mu }^{0},r_{\mu }^{0}\right)\in X_{\mu }\times W_{\mu }\times Q_{\mu }\times S_{\mu }$ in (33) and (34), we can access

$$\begin{array}{c} a_{s}\left(u_{\mu }^{0},u_{\mu }^{0}\right)+a_{m}\left(b_{\mu }^{0},b_{\mu }^{0}\right)+G\left(p_{\mu }^{0},p_{\mu }^{0}\right)+\frac{\sigma \mu }{R_{e}^{-1}}a_{s}\left(u_{\mu }^{0},u_{\mu }^{0}\right)=\langle f,u_{\mu }^{0}\rangle +\left(g,b_{\mu }^{0}\right), \end{array}$$

Using (20), we have

$$\begin{array}{c} C_{min}∥\left(u_{\mu }^{0},b_{\mu }^{0}\right){∥}_{1}\leq {∥F∥}_{*}. \end{array}$$

For m=J, assuming that (36) holds, it is sufficient to prove that it also holds for m=J+1. Let m=J+1 take $\left(v,c,q,s\right)=\left(u_{\mu }^{J+1},b_{\mu }^{J+1},p_{\mu }^{J+1},r_{\mu }^{J+1}\right)\in X_{\mu }\times W_{\mu }\times Q_{\mu }\times S_{\mu }$ in (33) and (34), using (8), we get

$$\begin{array}{c} a_{s}\left(u_{\mu }^{J+1},u_{\mu }^{J+1}\right)+a_{m}\left(b_{\mu }^{J+1},b_{\mu }^{J+1}\right)+G\left(p_{\mu }^{J+1},p_{\mu }^{J+1}\right)+\frac{\sigma \mu }{R_{e}^{-1}}a_{s}\left(u_{\mu }^{J+1},u_{\mu }^{J+1}\right)\\ =\left(f,u_{\mu }^{J+1}\right)+\left(g,b_{\mu }^{J+1}\right). \end{array}$$

and by applying (20) and (8), we derive that

$$\begin{array}{c} C_{min}∥\left(u_{\mu }^{J+1},b_{\mu }^{J+1}\right){∥}_{1}\leq {∥F∥}_{*}. \end{array}$$

Thus, the proof of the first part of (36) has been finished.

Applying (16) to (31), and using (10) in (32) with s=0, we have

$$\begin{array}{c} \begin{array}{cc} \; & \hat{\beta }\left(R_{e}^{-1}∥\nabla u_{\mu }^{m}{∥}_{0}+{∥p_{\mu }^{m}∥}_{0}\right)+\hat{\beta }{∥\nabla r_{\mu }^{m}∥}_{0}\\ \; & \;\leq C\hat{N}∥\left(u_{\mu }^{m-1},b_{\mu }^{m-1}\right){∥}_{1}{∥\left(u_{\mu }^{m},b_{\mu }^{m}\right)∥}_{1}\\ \; & \;\;+C\left(\sigma \mu C_{max}∥\left(u_{\mu }^{m},b_{\mu }^{m}\right){∥}_{1}+{∥F∥}_{*}\right). \end{array} \end{array}$$

Combining with the stability of $∥\left(u_{\mu }^{m},b_{\mu }^{m}\right){∥}_{1}$, we can complete the proof of (36). Similar to (27), (37) can be derived. The proof ends.    □

Next, we will establish the upper bound of error $\left(u_{\mu }-u_{\mu }^{m},b_{\mu }-b_{\mu }^{m},p_{\mu }-p_{\mu }^{m},r_{\mu }-r_{\mu }^{m}\right)$. For convenience, let $\left(e^{m},b^{m},\eta^{m},\tau^{m}\right)=\left(u_{\mu }-u_{\mu }^{m},b_{\mu }-b_{\mu }^{m},p_{\mu }-p_{\mu }^{m},r_{\mu }-r_{\mu }^{m}\right)$.

**Theorem** **2.** *Suppose that $0<\sigma_{1}<1$, for all $m\geq 0,\;(e^{m},b^{m},\eta^{m},\tau^{m})$ satisfies*

(38) $$\begin{array}{c} C_{min}∥\left(e^{m},b^{m}\right){∥}_{1}\leq \sigma_{1}^{m}{∥F∥}_{*},\;\;∥\left(\eta^{m},\tau^{m}\right)∥\leq C_{1}\sigma_{1}^{m}{∥F∥}_{*}, \end{array}$$

(39) $$\begin{array}{c} \begin{array}{c} ∥\left(u-u_{\mu }^{m},b-b_{\mu }^{m}\right){∥}_{1}+∥\left(p-p_{\mu }^{m},r-r_{\mu }^{m}\right)∥\leq C\mu^{\min\{1,\gamma ,\tau \}}+C\sigma_{1}^{m}. \end{array} \end{array}$$

**Proof.**  Subtracting (35) from (21), there holds

$$\begin{array}{c} A_{\mu }\left(e^{m},b^{m};v,c\right)+C\left(e^{m-1},b^{m-1};u_{\mu },b_{\mu };v,c\right)+C\left(u_{\mu }^{m-1},b_{\mu }^{m-1};e^{m},b^{m};v,c\right)\\ \;+G\left(\eta^{m},q\right)-B\left(\eta^{m},\tau^{m};v,c\right)+B\left(q,s;e^{m},b^{m}\right)=0. \end{array}$$

Choose $\left(v,c,q,s\right)=\left(e^{m},b^{m},\eta^{m},\tau^{m}\right)$ to obtain

(40) $$\begin{array}{c} A_{\mu }\left(e^{m},b^{m};e^{m},b^{m}\right)+C\left(e^{m-1},b^{m-1};u_{\mu },b_{\mu };e^{m},b^{m}\right)+G\left(\eta^{m},\eta^{m}\right)=0. \end{array}$$

It follows from (20), (9) and Lemma 2 that

$$\begin{array}{c} \begin{array}{cc} C_{min}{∥\left(e^{m},b^{m}\right)∥}_{1}\; & \leq \hat{N}∥\left(e^{m-1},b^{m-1}\right){∥}_{1}{∥\left(u_{\mu },b_{\mu }\right)∥}_{1}\\ \; & \leq \sigma_{1}C_{min}{∥\left(e^{m-1},b^{m-1}\right)∥}_{1}\\ \; & \leq \sigma_{1}^{m}C_{min}∥\left(e^{0},b^{0}\right){∥}_{1}\leq \sigma_{1}^{m}{∥F∥}_{*}. \end{array} \end{array}$$

Subtracting (31) from (16), we have

$$\begin{array}{c} \begin{array}{c} D_{\mu }\left(e^{m},\eta^{m};v,q\right)+c_{0}\left(e^{m-1},u_{\mu },v\right)+c_{0}\left(u_{\mu }^{m-1},e^{m},v\right)-c_{1}\left(b^{m-1},v,b_{\mu }\right)\\ \;+c_{1}\left(b_{\mu }^{m-1},v,b^{m}\right)+\frac{\sigma \mu }{R_{e}^{-1}}a_{s}\left(e^{m},v\right)=0. \end{array} \end{array}$$

Using the second equation of (17) minus equation (32) and choosing s=0, we get

$$\begin{array}{c} \begin{array}{c} b_{m}\left(\tau^{m},c\right)=a_{m}\left(b^{m},c\right)+c_{1}\left(b^{m-1},u_{\mu },c\right)+c_{1}\left(b_{\mu }^{m-1},e^{m},c\right). \end{array} \end{array}$$

Applying (16) and (10) to the above two equations, respectively, there holds

$$\begin{array}{c} \begin{array}{cc} \; & \hat{\beta }\left(R_{e}^{-1}∥\nabla e^{m}{∥}_{0}+{∥\eta^{m}∥}_{0}\right)+\hat{\beta }{∥\eta^{m}∥}_{0}\\ \; & \;\leq \hat{N}∥\left(e^{m-1},b^{m-1}\right){∥}_{1}{∥\left(u_{\mu },b_{\mu }\right)∥}_{1}\\ \; & \;\;+\hat{N}∥\left(u_{\mu }^{m-1},b_{\mu }^{m-1}\right){∥}_{1}{∥\left(e^{m},b^{m}\right)∥}_{1}\\ \; & \;\;+\sigma \mu ∥\left(e^{m},b^{m}\right){∥}_{1}\\ \; & \leq C\sigma_{1}^{m}{∥F∥}_{*}. \end{array} \end{array}$$

The result (39) can be obtained by (38) and Theorem 1. The proof ends.    □

## 3. Two-Level Stabilized Finite Element Algorithm

In this section, motivated by [38], two-level stabilized finite element algorithm for incompressible MHD equations is presented. The stability analysis and the optimal error estimation with respect to the mesh size *H* and *h* and the iterative step *m* are obtained.

It is evident that in Steps 2–5 of Algorithm 1 the iteration is controlled by $∥\left(u_{H}-u_{H}^{m},b_{H}-b_{H}^{m}\right){∥}_{1}\leq C{∥\left(u_{H}^{m}-u_{H}^{m-1},b_{H}^{m}-b_{H}^{m-1}\right)∥}_{0}$ (see Theorem 5 of [4]), which provides an operable way to acquire the desired solution $\left(u_{H}^{m},b_{H}^{m}\right)$.
**Algorithm 1** Two-level stabilized finite element algorithm1:Give the initial value $\left(u_{H}^{0},b_{H}^{0},p_{H}^{0},r_{H}^{0}\right)\in X_{H}\times W_{H}\times Q_{H}\times S_{H}$ by (33) and (34) with μ=H.2:**while**$∥\left(u_{H}^{m}-u_{H}^{m-1},b_{H}^{m}-b_{H}^{m-1}\right){∥}_{0}>\epsilon$**do**3:    Solve MHD on the coarse grid: Find $\left(u_{H}^{m},b_{H}^{m},p_{H}^{m},r_{H}^{m}\right)\in X_{H}\times W_{H}\times Q_{H}\times S_{H}$ by (35) with μ=H.4:    $\left(u_{H}^{m-1},b_{H}^{m-1},p_{H}^{m-1},r_{H}^{m-1}\right)=\left(u_{H}^{m},b_{H}^{m},p_{H}^{m},r_{H}^{m}\right).$5:**end while**6:Find $\left(u_{mh},b_{mh},p_{mh},r_{mh}\right)\in X_{h}\times W_{h}\times Q_{h}\times S_{h}$ on the fine grid, for any $\left(v,c,q,s\right)\in X_{h}\times W_{h}\times Q_{h}\times S_{h}$, such that
(41)$$\begin{array}{c} a_{s}\left(u_{mh},v\right)+c_{0}\left(u_{H}^{m},u_{mh},v\right)-c_{1}\left(b_{H}^{m},v,b_{mh}\right)-b_{s}\left(p_{mh},v\right)+b_{s}\left(q,u_{mh}\right)\\ +G\left(p_{mh},q\right)+\frac{\sigma h}{R_{e}^{-1}}a_{s}\left(u_{mh},v\right)=\langle f,v\rangle , \end{array}$$
(42)$$\begin{array}{c} a_{m}\left(b_{mh},c\right)+c_{1}\left(b_{H}^{m},u_{mh},c\right)-b_{m}\left(r_{mh},c\right)+b_{m}\left(s,b_{mh}\right)=\left(g,c\right). \end{array}$$

**Theorem** **3.** *Under the assumption of Theorem 2, the solutions $\left(u_{mh},b_{mh}\right)$ obtained from (41) and (42) satisfy*

(43) $$\begin{array}{c} C_{min}∥\left(u_{mh},b_{mh}\right){∥}_{1}\leq {∥F∥}_{*}, \end{array}$$

(44) $$\begin{array}{c} \begin{array}{c} ∥\left(u-u_{mh},b-b_{mh}\right){∥}_{1}+∥\left(p-p_{mh},r-r_{mh}\right)∥\\ \;\leq C(H^{2}+H^{2\min\{1,\gamma ,\tau \}}+H^{\min\{2,\gamma +1,\tau +1\}}+h+h^{\min\{1,\gamma ,\tau \}}+\sigma_{1}^{m}). \end{array} \end{array}$$

**Proof.**  Taking $\left(v,c,q,s\right)=\left(u_{mh},b_{mh},p_{mh},r_{mh}\right)\in X_{h}\times W_{h}\times Q_{h}\times S_{h}$ in (41) and (42), by using of (8) and (20), we can easily derive (43).

Subtracting (41) and (42) from (2) and (3), respectively, we have

(45) $$\begin{array}{c} A\left(u-u_{mh},b-b_{mh};v,c\right)+C\left(u-u_{H}^{m},b-b_{H}^{m};u,b;v,c\right)\\ \;+C\left(u_{H}^{m},b_{H}^{m};u-u_{mh},b-b_{mh};v,c\right)-B\left(p-p_{mh},r-r_{mh};v,c\right)\\ \;+B\left(q,s;u-u_{mh},b-b_{mh}\right)-G\left(p_{mh},q\right)=\frac{\sigma h}{R_{e}^{-1}}a_{s}\left(u_{mh},v\right). \end{array}$$

By the two projection operators of (22) and (24) with μ=h, letting $e_{uh}=R\left(u,p\right)-u_{mh},e_{bh}=\Lambda b-b_{mh},e_{ph}=Q\left(u,p\right)-p_{mh},e_{rh}=\Lambda r-r_{mh}$, and then taking $v=e_{uh},c=e_{bh},q=e_{ph},s=e_{rh}$, there holds

(46) $$\begin{array}{c} A_{\mu }\left(e_{uh},e_{bh};e_{uh},e_{bh}\right)+G\left(e_{ph},e_{ph}\right)\\ \;=C(u_{H}^{m}-u,b_{H}^{m}-b;u-u_{H}^{m},b-b_{H}^{m};e_{uh},e_{bh})\\ \;+C(u_{H}^{m}-u,b_{H}^{m}-b;u_{H}^{m},b_{H}^{m};e_{uh},e_{bh})\\ \;+C(u_{H}^{m},b_{H}^{m};R\left(u,p\right)-u,\Lambda b-b;e_{uh},e_{bh})\\ \;+\frac{\sigma h}{R_{e}^{-1}}a_{s}\left(u,e_{uh}\right)+\frac{\sigma h}{R_{e}^{-1}}a_{s}\left(R\left(u,p\right)-u,e_{uh}\right)\\ \;+G\left(p,e_{ph}\right)+G\left(Q\left(u,p\right)-p,e_{ph}\right). \end{array}$$

The left-hand side of (46) can be estimated as

(47) $$\begin{array}{c} \begin{array}{cc} l.h.s\; & \geq C_{min}∥\left(e_{uh},e_{bh}\right){∥}_{1}^{2}+{∥e_{ph}-\Pi_{h}e_{ph}∥}_{0}^{2}\\ \; & \geq \min\left\{C_{min},1\right\}\left(∥\left(e_{uh},e_{bh}\right){∥}_{1}^{2}+{∥e_{ph}-\Pi_{h}e_{ph}∥}_{0}^{2}\right). \end{array} \end{array}$$

Making use of (14), the right-hand side of (46) for G(·,·) can be estimated as

(48) $$\begin{array}{c} \begin{array}{cc} G(p,e_{ph}) & =(p-\Pi_{h}p,e_{ph}-\Pi_{h}e_{ph})\\ \; & \leq Ch^{\min\{1,\;\gamma \}}{∥p∥}_{\gamma }{∥e_{ph}-\Pi_{h}e_{ph}∥}_{0}, \end{array} \end{array}$$

(49) $$\begin{array}{c} \begin{array}{cc} G(Q\left(u,p\right)-p,e_{ph}) & \leq {C∥Q\left(u,p\right)-p∥}_{0}{∥e_{ph}-\Pi_{h}e_{ph}∥}_{0}\\ \; & \leq Ch^{\min\{1,\gamma \}}{(∥u∥}_{1+\gamma }+{∥p∥}_{\gamma })∥e_{ph}-\Pi_{h}e_{ph}{∥}_{0}. \end{array} \end{array}$$

Using (9), (12) and (37), as well as Theorems 1, 2 and Lemma 2, the right-hand side of (46) can be estimated that

(50) $$\begin{array}{cc} r.h.s\; & \leq \hat{N}∥\left(u-u_{H}^{m},b-b_{H}^{m}\right){∥}_{1}^{2}{∥\left(e_{uh},e_{bh}\right)∥}_{1}\\ \; & \;+\hat{N}∥\left(u-u_{H},b-b_{H}\right){∥}_{0}∥\left(A_{1H}u_{H}^{m},A_{2H}b_{H}^{m}\right){∥}_{0}{∥\left(e_{uh},e_{bh}\right)∥}_{1}\\ \; & \;+\hat{N}∥\left(u_{H}-u_{H}^{m},b_{H}-b_{H}^{m}\right){∥}_{1}∥\left(u_{H}^{m},b_{H}^{m}\right){∥}_{1}{∥\left(e_{uh},e_{bh}\right)∥}_{1}\\ \; & \;+\hat{N}∥\left(u_{H}^{m},b_{H}^{m}\right){∥}_{1}∥\left(u-R\left(u,p\right),\;b-\Lambda b\right){∥}_{1}{∥\left(e_{uh},e_{bh}\right)∥}_{1}\\ \; & \;+{\sigma h∥\left(u,b\right)∥}_{1}{∥\left(e_{uh},e_{bh}\right)∥}_{1}\\ \; & \;+{\sigma h∥\left(u-R\left(u,\;p\right),\;b-\Lambda b\right)∥}_{1}{∥\left(e_{uh},e_{bh}\right)∥}_{1}\\ \; & \;+Ch^{\min\{1,\gamma \}}{(∥u∥}_{1+\gamma }+{∥p∥}_{\gamma })∥e_{ph}-\Pi_{h}e_{ph}{∥}_{0}.\\ \; & \leq C(H^{2\min\{1,\gamma ,\tau \}}+\sigma_{1}^{2m}+CH^{\min\{2,\gamma +1,\tau +1\}}+H^{2}+\sigma_{1}^{m}+h+h^{\min\{1,\;\gamma ,\;\tau \}})\\ \; & \;\;\cdot {\left(∥\left(e_{uh},e_{bh}\right){∥}_{1}^{2}+{∥e_{ph}-\Pi_{h}e_{ph}∥}_{0}^{2}\right)}^{\frac{1}{2}}, \end{array}$$

Combining (23), (25) and (47) with (50), we can get the first part of (44).

To estimate the pressure, we rewrite (45) with s=0 as

$$\begin{array}{c} \begin{array}{c} D_{\mu }\left(u-u_{mh},p-p_{mh};v,q\right)+c_{0}\left(u-u_{H}^{m},u,v\right)+c_{0}\left(u_{H}^{m},u-u_{mh},v\right)\\ \;-c_{1}\left(b-b_{H}^{m},v,b\right)-c_{1}\left(b_{H}^{m},v,b-b_{mh}\right)-\frac{\sigma h}{R_{e}^{-1}}a_{s}\left(u_{mh},v\right)=G\left(p,q\right), \end{array} \end{array}$$

$$\begin{array}{c} b_{m}\left(r-r_{mh},c\right)=a_{m}\left(b-b_{mh},c\right)+c_{1}\left(b-b_{H}^{m},u-u_{H}^{m},c\right)+c_{1}\left(b-b_{H}^{m},u_{H}^{m},c\right)\\ \;+c_{1}\left(b_{H}^{m},u-u_{H}^{m},c\right). \end{array}$$

Applying (16), (10) and the standard technique to the above two equations, we can derive the second part of (44). We complete the proof.   □

## 4. Numerical Examples

In this section, some numerical experiments are shown to verify the correctness and effectiveness of the one-level stabilized finite element method and the two-level stabilized one. Here, the velocity, pressure and quasi-pressure are approximated by $P_{1}$ and the magnetic field by the first (or second) class Nédélec edge element. SFEM denotes by the stabilized finite element method (31) and (32). The software FEAlPy V1.0 [39] created by Huayi Wei, Xiangtan University, Xiangtan, China is used in the numerical examples.

**Smooth solution in 2D**: Set $\Omega ={\left[0,\;1\right]}^{2}$ and $R_{e}=R_{m}=S=1,\sigma =0.01$. Given the source terms f,g such that the exact solution is

$$\left\{\begin{array}{cc} u_{1} & =10x_{1}^{2}{\left(x_{1}-1\right)}^{2}x_{2}\left(x_{2}-1\right)\left(2x_{2}-1\right),\\ u_{2} & =-10x_{1}\left(x_{1}-1\right)\left(2x_{1}-1\right)x_{2}^{2}{\left(x_{2}-1\right)}^{2},\\ b_{1} & =\cos\left(\pi x_{1}\right)\sin\left(\pi x_{2}\right),\\ b_{2} & =-\sin\left(\pi x_{1}\right)\cos\left(\pi x_{2}\right),\\ p & =10\left(2x_{1}-1\right)\left(2x_{2}-1\right),\\ r & =0. \end{array}\right.$$

Table 1 and Table 2 display the errors of SFEM and two-level SFEM for 2D MHD Equation (1). It is shown that the corresponding errors are smaller and smaller along with the grid getting finer and finer, the convergence order is optimal. When $h=O(H^{2})$, the error accuracy of the two methods is almost the same. From CPU time, compared to SFEM, two-level SFEM save much computational cost.

The numerical results are listed in Table 3 and Table 4 when the magnetic field is approximated by the second class Nédélec element. Clearly, the convergence order of $||\;b-{\;b}_{h}{\left|\right|}_{0}$ is one higher than that in Table 1 and Table 2, which is consistent with the general theoretical analysis results of the Nédélec element.

**Smooth solution in 3D:** Set $\Omega ={\left[0,\;1\right]}^{3}$ and $R_{e}=R_{m}=S=1,\sigma =0.01$. Given f,g such that the exact solution is:

$$\left\{\begin{array}{cc} u_{1} & =0.5\sin\left(\pi x_{1}\right)cos\left(\pi x_{2}\right)cos\left(\pi x_{3}\right),\\ u_{2} & =0.5\cos\left(\pi x_{1}\right)sin\left(\pi x_{2}\right)cos\left(\pi x_{3}\right),\\ u_{3} & =-\cos\left(\pi x_{1}\right)cos\left(\pi x_{2}\right)sin\left(\pi x_{3}\right),\\ b_{1} & =0.5\cos\left(\pi x_{1}\right)\sin\left(\pi x_{2}\right)\sin\left(\pi x_{3}\right),\\ b_{2} & =-\sin\left(\pi x_{1}\right)\cos\left(\pi x_{2}\right)\sin\left(\pi x_{3}\right),\\ b_{3} & =0.5\sin\left(\pi x_{1}\right)\sin\left(\pi x_{2}\right)\cos\left(\pi x_{3}\right),\;\\ p & =\cos\left(\pi x_{1}\right)\cos\left(\pi x_{2}\right)\cos\left(\pi x_{3}\right),\\ r & =0. \end{array}\right.$$

**Figure 1:**
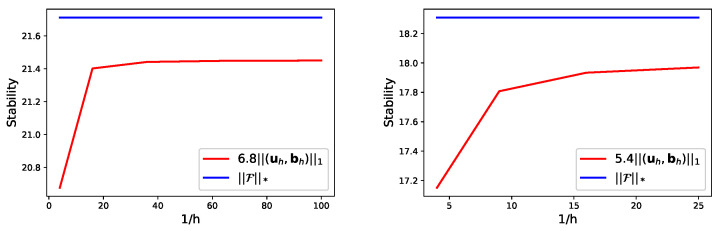
Stability of 2D (**left**) and 3D (**right**) problems.

In Table 5 and Table 6, the variable quantity (u,b,p,r) is approximated by $P_{1}$, the first class Nédélec element, $P_{1}$ and $P_{1}$, respectively. Table 7 and Table 8 list the results when (u,b,p,r) is approximated by $P_{1}$, the second class Nédélec element, $P_{1}$ and $P_{1}$. It is observed that the numerical results agree well with the theoretical results of Theorems 1–3. On the other hand, from Table 7 and Table 8, we find that SFEM (35) does not work when H=1/16, however two-level SFEM (41) and (42) is valid in the current computing environment for our computer. On the other hand, the stability results of (43) are checked by Figure 1.

**2D MHD problem with a singular solution:** We consider 2D MHD system (1) in the L-type domain $\Omega :={\left[-1,\;1\right]}^{2}\backslash \left(\left[0,\;1\right]\times \left[-1,\;0\right]\right)$. Set $R_{e}=S=1,R_{m}=0.001$, the analytical solution in polar coordinates (ρ,φ) is given by [40]

$$\left\{\begin{array}{cc} u(\rho ,\varphi ) & =\left[\begin{array}{c} \rho^{\lambda }\left(\left(1+\lambda \right)\sin\left(\varphi \right)\psi \left(\phi \right)+\cos\left(\varphi \right)\psi^{\prime }\left(\varphi \right)\right)\\ \rho^{\lambda }\left(-\left(1+\lambda \right)\cos\left(\varphi \right)\psi \left(\varphi \right)+\sin\left(\varphi \right)\psi^{\prime }\left(\varphi \right)\right) \end{array}\right],\\ b(\rho ,\;\varphi ) & =\nabla \left(\rho^{2/3}\sin\left(2/3\varphi \right)\right),\\ p(\rho ,\varphi ) & =-\rho^{\lambda -1}\left({\left(1+\lambda \right)}^{2}\psi^{\prime }\left(\varphi \right)+\psi^{\prime \prime \prime }\left(\varphi \right)\right)/\left(1-\lambda \right),\\ r & =0,\\ \psi (\varphi ) & =\sin((1+\lambda )\varphi )\cos(\lambda \omega )/(1+\lambda )-\cos((1+\lambda )\varphi )\\ \; & -\sin((1-\lambda )\varphi )\cos(\lambda \omega )/(1-\lambda )+\cos((1-\lambda )\varphi ),\\ \omega & =\frac{3}{2}\pi ,\\ \lambda & \approx 0.54448373678246. \end{array}\right.$$

In the 2D case, there holds the regularity $\;u\in H^{1+\lambda }\left(\Omega \right)$, $\;b\in H^{\frac{2}{3}}\left(\Omega \right)$ and $p\in H^{\lambda }\left(\Omega \right)$.

**Figure 2:**
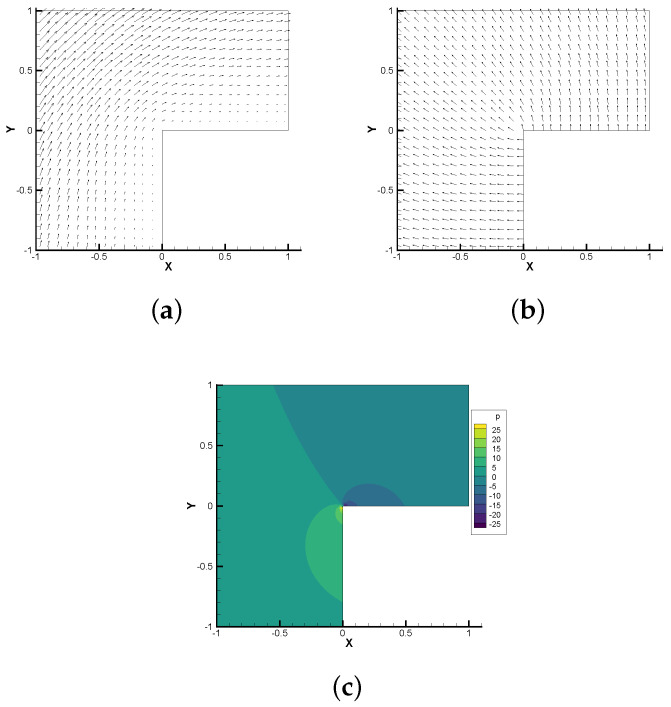
Numerical approximations of 2D singular solution. (**a**) velocity field; (**b**) magnetic field; (**c**) pressure.

In Table 9 and Table 10, (u,b,p,r) is approximated by $P_{1}$, the second class Nédélec element, $P_{1}$ and $P_{1}$. Because the regularity of velocity, magnetic field and pressure is low, the convergence of $||\;u-{\;u}_{h}{\left|\right|}_{0}$, $||\nabla \left(\;u-{\;u}_{h}\right){\left|\right|}_{0}$, $||p-p_{h}{\left|\right|}_{0}$, $||\;b-{\;b}_{h}{\left|\right|}_{0}$, $||\;b-{\;b}_{h}{\left|\right|}_{\mathrm{curl}}$ keep the rate of 1.4, 0.54, 0.59, 0.66, 0.66, respectively, which verify the correctness of the theoretical analysis (Theorems 2 and 3) results. In Figure 2 we display the streamlines of the velocity field and magnetic field, and the contours of the pressure, which are consistent with the numerical results in the literature [40].

## 5. Conclusions

Based on the stabilization and the Lagrange multiplier techniques, the stabilized finite element algorithm is designed for the stationary incompressible MHD. The Lagrange multiplier technique idea helps us in dealing with the low regular magnetic field sub-problem by H(curl;Ω)-conforming element. The stabilized one by using local Gauss integration allow us to adopt the lowest equal-order elements to approximate the flow field sub-problem. The stability and optimal convergence analysis are given. Furthermore, the two-level stabilized finite element algorithms are presented. In the first step we combine the stabilized finite element method with the Oseen iteration for the nonlinear MHD equations on a coarse grid. For the second step, we employ the linearized correction on a fine grid. We give the optimal error analysis, which shows that when the grid sizes satisfy $h=O(H^{2})$, the two-level stabilization method not only has the optimal convergence order, but also can save more computational cost than the one-level method. These theoretical analysis results have been verified by some numerical experiments.

## Figures and Tables

**Table 1:** Convergence of ${\;u}_{h}$ and $p_{h}$ (first class Nédélec element).

*h*	*H*	$||\;u-{\;u}_{h}{\left|\right|}_{0}$	order	$||\nabla \left(\;u-{\;u}_{h}\right){\left|\right|}_{0}$	order	$||p-p_{h}{\left|\right|}_{0}$	order	CPU(s)
SFEM	1/16	4.31 $\times \;10^{-3}$		7.14 $\times \;10^{-2}$		1.19 $\times \;10^{-1}$		0.49
1/16	1/4	4.28 $\times \;10^{-3}$		7.15 $\times \;10^{-2}$		1.28 $\times \;10^{-1}$		0.36
SFEM	1/36	8.80 $\times \;10^{-4}$	1.96	2.62 $\times \;10^{-2}$	1.24	2.93 $\times \;10^{-2}$	1.73	2.79
1/36	1/6	8.76 $\times \;10^{-4}$	1.96	2.64 $\times \;10^{-2}$	1.23	3.36 $\times \;10^{-2}$	1.65	1.70
SFEM	1/64	2.81 $\times \;10^{-4}$	1.98	1.36 $\times \;10^{-2}$	1.13	1.10 $\times \;10^{-2}$	1.71	11.13
1/64	1/8	2.81 $\times \;10^{-4}$	1.98	1.38 $\times \;10^{-2}$	1.13	1.36 $\times \;10^{-2}$	1.57	6.16
SFEM	1/100	1.15 $\times \;10^{-4}$	1.99	8.43 $\times \;10^{-3}$	1.08	5.21 $\times \;10^{-3}$	1.67	35.79
1/100	1/10	1.16 $\times \;10^{-4}$	1.97	8.53 $\times \;10^{-3}$	1.08	6.99 $\times \;10^{-3}$	1.49	17.78

**Table 2:** Convergence of ${\;b}_{h}$ and $r_{h}$ (first class Nédélec element).

*h*	*H*	$||\;b-{\;b}_{h}{\left|\right|}_{0}$	order	$||\;b-{\;b}_{h}{\left|\right|}_{\mathrm{curl}}$	order	$||r-r_{h}{\left|\right|}_{0}$	CPU(s)
SFEM	1/16	4.01 $\times \;10^{-2}$		2.09 $\times \;10^{-1}$		1.22 $\times \;10^{-14}$	0.49
1/16	1/4	4.01 $\times \;10^{-2}$		2.09 $\times \;10^{-1}$		1.42 $\times \;10^{-14}$	0.36
SFEM	1/36	1.78 $\times \;10^{-2}$	1.00	9.30 $\times \;10^{-2}$	1.00	5.20 $\times \;10^{-14}$	2.79
1/36	1/6	1.78 $\times \;10^{-2}$	1.00	9.31 $\times \;10^{-2}$	1.00	5.03 $\times \;10^{-14}$	1.70
SFEM	1/64	1.00 $\times \;10^{-2}$	1.00	5.23 $\times \;10^{-2}$	1.00	1.33 $\times \;10^{-13}$	11.13
1/64	1/8	1.00 $\times \;10^{-2}$	1.00	5.24 $\times \;10^{-2}$	1.00	1.43 $\times \;10^{-13}$	6.16
SFEM	1/100	6.41 $\times \;10^{-3}$	1.00	3.35 $\times \;10^{-2}$	1.00	2.28 $\times \;10^{-13}$	35.79
1/100	1/10	6.41 $\times \;10^{-3}$	1.00	3.35 $\times \;10^{-2}$	1.00	2.27 $\times \;10^{-13}$	17.78

**Table 3:** Convergence of ${\;u}_{h}$ and $p_{h}$ (second class Nédélec element).

*h*	*H*	$||\;u-{\;u}_{h}{\left|\right|}_{0}$	order	$||\nabla \left(\;u-{\;u}_{h}\right){\left|\right|}_{0}$	order	$||p-p_{h}{\left|\right|}_{0}$	order	CPU(s)
SFEM	1/16	4.31 $\times \;10^{-3}$		7.14 $\times \;10^{-2}$		1.20 $\times \;10^{-1}$		0.78
1/16	1/4	4.28 $\times \;10^{-3}$		7.15 $\times \;10^{-2}$		1.28 $\times \;10^{-1}$		0.47
SFEM	1/36	8.80 $\times \;10^{-4}$	1.96	2.62 $\times \;10^{-2}$	1.24	2.94 $\times \;10^{-2}$	1.73	7.63
1/36	1/6	8.76 $\times \;10^{-4}$	1.96	2.64 $\times \;10^{-2}$	1.23	3.36 $\times \;10^{-2}$	1.65	3.33
SFEM	1/64	2.81 $\times \;10^{-4}$	1.98	1.36 $\times \;10^{-2}$	1.13	1.10 $\times \;10^{-2}$	1.70	48.23
1/64	1/8	2.81 $\times \;10^{-4}$	1.98	1.38 $\times \;10^{-2}$	1.13	1.36 $\times \;10^{-2}$	1.57	18.00
SFEM	1/100	1.15 $\times \;10^{-4}$	1.99	8.43 $\times \;10^{-3}$	1.08	5.59 $\times \;10^{-3}$	1.53	205.89
1/100	1/10	1.16 $\times \;10^{-4}$	1.98	8.53 $\times \;10^{-3}$	1.08	6.99 $\times \;10^{-3}$	1.49	71.25

**Table 4:** Convergence of ${\;b}_{h}$ and $r_{h}$ (second class Nédélec element).

*h*	*H*	$||\;b-{\;b}_{h}{\left|\right|}_{0}$	order	$||\;b-{\;b}_{h}{\left|\right|}_{\mathrm{curl}}$	order	$||r-r_{h}{\left|\right|}_{0}$	CPU(s)
SFEM	1/16	4.19 $\times \;10^{-3}$		2.05 $\times \;10^{-1}$		1.44 $\times \;10^{-14}$	0.78
1/16	1/4	4.10 $\times \;10^{-3}$		2.05 $\times \;10^{-1}$		1.78 $\times \;10^{-14}$	0.47
SFEM	1/36	8.33 $\times \;10^{-4}$	1.99	9.13 $\times \;10^{-2}$	1.00	6.62 $\times \;10^{-14}$	7.63
1/36	1/6	8.39 $\times \;10^{-4}$	1.98	9.31 $\times \;10^{-2}$	1.00	7.60 $\times \;10^{-14}$	3.33
SFEM	1/64	2.63 $\times \;10^{-4}$	2.00	5.14 $\times \;10^{-2}$	1.00	1.49 $\times \;10^{-13}$	48.23
1/64	1/8	2.71 $\times \;10^{-4}$	1.96	5.14 $\times \;10^{-2}$	1.00	1.74 $\times \;10^{-13}$	18.00
SFEM	1/100	1.08 $\times \;10^{-4}$	1.99	3.28 $\times \;10^{-2}$	1.00	2.71 $\times \;10^{-13}$	205.89
1/100	1/10	1.15 $\times \;10^{-4}$	1.91	3.29 $\times \;10^{-2}$	1.00	3.85 $\times \;10^{-13}$	71.25

**Table 5:** Convergence of ${\;u}_{h}$ and $p_{h}$ (first class Nédélec element).

*h*	*H*	$||\;u-{\;u}_{h}{\left|\right|}_{0}$	order	$||\nabla \left(\;u-{\;u}_{h}\right){\left|\right|}_{0}$	order	$||p-p_{h}{\left|\right|}_{0}$	order	CPU(s)
SFEM	1/4	7.86 $\times \;10^{-2}$		1.17		1.39		2.03
1/4	1/2	7.86 $\times \;10^{-2}$		1.17		1.39		1.44
SFEM	1/9	1.67 $\times \;10^{-2}$	1.91	5.33 $\times \;10^{-1}$	0.97	3.26 $\times \;10^{-1}$	1.79	28.05
1/9	1/3	1.67 $\times \;10^{-2}$	1.91	5.34 $\times \;10^{-1}$	0.97	3.29 $\times \;10^{-1}$	1.78	16.26
SFEM	1/16	5.34 $\times \;10^{-3}$	1.98	2.99 $\times \;10^{-1}$	1.00	1.08 $\times \;10^{-1}$	1.91	577.84
1/16	1/4	5.38 $\times \;10^{-3}$	1.98	2.99 $\times \;10^{-1}$	1.00	1.12 $\times \;10^{-1}$	1.86	189.50

**Table 6:** Convergence of ${\;b}_{h}$ and $r_{h}$ (first class Nédélec element).

*h*	*H*	$||\;b-{\;b}_{h}{\left|\right|}_{0}$	order	$||\;b-{\;b}_{h}{\left|\right|}_{\mathrm{curl}}$	order	$||r-r_{h}{\left|\right|}_{0}$	CPU(s)
SFEM	1/4	1.38 $\times \;10^{-1}$		8.38 $\times \;10^{-1}$		7.86 $\times \;10^{-16}$	2.03
1/4	1/2	1.38 $\times \;10^{-1}$		8.39 $\times \;10^{-1}$		9.82 $\times \;10^{-16}$	1.44
SFEM	1/9	6.16 $\times \;10^{-2}$	1.00	3.81 $\times \;10^{-1}$	0.97	4.24 $\times \;10^{-15}$	28.05
1/9	1/3	6.17 $\times \;10^{-2}$	0.99	3.83 $\times \;10^{-1}$	0.96	4.71 $\times \;10^{-15}$	16.26
SFEM	1/16	3.47 $\times \;10^{-2}$	1.00	2.14 $\times \;10^{-1}$	1.00	1.59 $\times \;10^{-14}$	577.84
1/16	1/4	3.47 $\times \;10^{-2}$	1.00	2.18 $\times \;10^{-1}$	0.98	1.42 $\times \;10^{-14}$	189.50

**Table 7:** Convergence of $u_{h}$ and $p_{h}$ (second class Nédélec element).

*h*	*H*	$||\;u-{\;u}_{h}{\left|\right|}_{0}$	order	$||\nabla \left(\;u-{\;u}_{h}\right){\left|\right|}_{0}$	order	$||p-p_{h}{\left|\right|}_{0}$	order	CPU(s)
SFEM	1/4	7.86 $\times \;10^{-2}$		1.17		1.39		2.39
1/4	1/2	7.86 $\times \;10^{-2}$		1.17		1.40		1.85
SFEM	1/9	1.67 $\times \;10^{-2}$	1.91	5.33 $\times \;10^{-1}$	0.97	3.26 $\times \;10^{-1}$	1.79	99.42
1/9	1/3	1.67 $\times \;10^{-2}$	1.91	5.34 $\times \;10^{-1}$	0.97	3.30 $\times \;10^{-1}$	1.78	42.96
SFEM	1/16	\	\	\	\	\	\	\
1/16	1/4	5.35 $\times \;10^{-3}$	1.98	2.99 $\times \;10^{-1}$	1.00	1.11 $\times \;10^{-1}$	1.86	2877.53

**Table 8:** Convergence of ${\;b}_{h}$ and $r_{h}$ (second class Nédélec element).

*h*	*H*	$||\;b-{\;b}_{h}{\left|\right|}_{0}$	order	$||\;b-{\;b}_{h}{\left|\right|}_{\mathrm{curl}}$	order	$||r-r_{h}{\left|\right|}_{0}$	CPU(s)
SFEM	1/4	6.64 $\times \;10^{-2}$		8.29 $\times \;10^{-1}$		8.89 $\times \;10^{-16}$	2.39
1/4	1/2	6.64 $\times \;10^{-2}$		8.30 $\times \;10^{-1}$		1.10 $\times \;10^{-15}$	1.85
SFEM	1/9	1.40 $\times \;10^{-2}$	1.91	3.76 $\times \;10^{-1}$	0.98	5.60 $\times \;10^{-15}$	99.42
1/9	1/3	1.41 $\times \;10^{-2}$	1.91	3.76 $\times \;10^{-1}$	0.97	6.19 $\times \;10^{-15}$	42.96
SFEM	1/16	\	\	\	\	\	\
1/16	1/4	4.61 $\times \;10^{-3}$	1.95	2.12 $\times \;10^{-1}$	1.00	2.26 $\times \;10^{-14}$	2877.53

**Table 9:** Convergence of ${\;u}_{h}$ and $p_{h}$.

*h*	*H*	$||\;u-{\;u}_{h}{\left|\right|}_{0}$	order	$||\nabla \left(\;u-{\;u}_{h}\right){\left|\right|}_{0}$	order	$||p-p_{h}{\left|\right|}_{0}$	order	CPU(s)
SFEM	1/4	9.97 $\times \;10^{-2}$		1.55		1.74		0.16
1/4	1/2	9.96 $\times \;10^{-2}$		1.55		1.76		0.16
SFEM	1/16	1.27 $\times \;10^{-2}$	1.48	7.41 $\times \;10^{-1}$	0.54	7.74 $\times \;10^{-1}$	0.59	1.89
1/16	1/4	1.29 $\times \;10^{-2}$	1.47	7.41 $\times \;10^{-1}$	0.54	7.80 $\times \;10^{-1}$	0.59	1.11
SFEM	1/36	3.95 $\times \;10^{-3}$	1.45	4.78 $\times \;10^{-1}$	0.54	5.00 $\times \;10^{-1}$	0.54	12.03
1/36	1/6	4.06 $\times \;10^{-3}$	1.43	4.78 $\times \;10^{-1}$	0.54	5.22 $\times \;10^{-1}$	0.49	6.04
SFEM	1/64	1.77 $\times \;10^{-3}$	1.39	3.50 $\times \;10^{-1}$	0.54	3.76 $\times \;10^{-1}$	0.49	49.61
1/64	1/8	1.84 $\times \;10^{-3}$	1.37	3.50 $\times \;10^{-1}$	0.54	3.93 $\times \;10^{-1}$	0.49	22.80

**Table 10:** Convergence of ${\;b}_{h}$ and $r_{h}$.

*h*	*H*	$||\;b-{\;b}_{h}{\left|\right|}_{0}$	order	$||\;b-{\;b}_{h}{\left|\right|}_{\mathrm{curl}}$	order	$||r-r_{h}{\left|\right|}_{0}$	CPU(s)
SFEM	1/4	1.91 $\times \;10^{-1}$		1.91 $\times \;10^{-1}$		5.98 $\times \;10^{-4}$	0.16
1/4	1/2	1.90 $\times \;10^{-1}$		1.91 $\times \;10^{-1}$		5.98 $\times \;10^{-4}$	0.16
SFEM	1/16	7.88 $\times \;10^{-2}$	0.64	7.88 $\times \;10^{-2}$	0.64	1.47 $\times \;10^{-4}$	1.89
1/16	1/4	7.88 $\times \;10^{-2}$	0.64	7.88 $\times \;10^{-2}$	0.64	1.47 $\times \;10^{-4}$	1.11
SFEM	1/36	4.63 $\times \;10^{-2}$	0.65	4.63 $\times \;10^{-2}$	0.65	5.84 $\times \;10^{-5}$	12.03
1/36	1/6	4.63 $\times \;10^{-2}$	0.65	4.63 $\times \;10^{-2}$	0.65	5.84 $\times \;10^{-5}$	6.04
SFEM	1/64	3.17 $\times \;10^{-2}$	0.66	3.17 $\times \;10^{-2}$	0.66	2.97 $\times \;10^{-5}$	49.61
1/64	1/8	3.17 $\times \;10^{-2}$	0.66	3.17 $\times \;10^{-2}$	0.66	2.97 $\times \;10^{-5}$	22.80

## Data Availability

Not applicable.

## Appendix A. Proof of Theorem 1

In this part, we will give the detail proof of Theorem 1.

**Proof.** Let $e_{u}=R\left(u,p\right)-u_{\mu },e_{b}=\wedge b-b_{\mu },e_{p}=Q\left(u,p\right)-p_{\mu },e_{r}=\wedge r-r_{\mu }.$ Subtract (18) from (4) and choose $v=e_{u},c=e_{b},q=e_{p},s=e_{r}$, we get

(A1) $$\begin{array}{c} A_{\mu }\left(e_{u},e_{b};e_{u},e_{b}\right)+C\left(e_{u},e_{b};u,b;e_{u},e_{b}\right)+G\left(e_{p},e_{p}\right)\\ \;=C(R\left(u,p\right)-u,\wedge b-b;u,b;e_{u},e_{b})\\ \;+C(u_{\mu },b_{\mu };R\left(u,p\right)-u,\wedge b-b;e_{u},e_{b})\\ \;+\frac{\sigma \mu }{R_{e}^{-1}}a_{s}\left(u,e_{u}\right)+\frac{\sigma \mu }{R_{e}^{-1}}a_{s}\left(R\left(u,p\right)-u,e_{u}\right)\\ \;+G\left(p,e_{p}\right)+G\left(Q\left(u,p\right)-p,e_{p}\right). \end{array}$$

Using (9) and (20) and Cauchy-Schwarz inequality, the left-hand side of (A1) can be bounded by

(A2) $$\begin{array}{cc} l.h.s.\; & \geq \left(C_{min}-\frac{\hat{N}{∥F∥}_{*}}{\underline{v}}\right)∥\left(e_{u},e_{b}\right){∥}_{1}^{2}+{∥e_{p}-\Pi_{\mu }e_{p}∥}_{0}^{2}\\ \; & \geq \underline{v}\left(1-\sigma_{0}\right)∥\left(e_{u},e_{b}\right){∥}_{1}^{2}+{∥e_{p}-\Pi_{\mu }e_{p}∥}_{0}^{2}\\ \; & \geq \min\left\{\underline{v}\left(1-\sigma_{0}\right),1\right\}\left(∥\left(e_{u},e_{b}\right){∥}_{1}^{2}+{∥e_{p}-\Pi_{\mu }e_{p}∥}_{0}^{2}\right), \end{array}$$

where $\sigma_{0}=\frac{\hat{N}{∥F∥}_{*}}{{\underline{v}}^{2}}.$ By (14), the two terms of the right-hand side of (A1) with respect to G(·,·) can be estimated as

(A3) $$\begin{array}{c} \begin{array}{cc} G(p,e_{p}) & \leq ∥p-\Pi_{\mu }{p∥}_{0}{∥e_{p}-\Pi_{\mu }e_{p}∥}_{0}\\ \; & \leq C\mu^{\min\{1,\gamma \}}{∥p∥}_{\gamma }{∥e_{p}-\Pi_{\mu }e_{p}∥}_{0}, \end{array} \end{array}$$

(A4) $$\begin{array}{c} \begin{array}{cc} G(Q\left(u,p\right)-p,e_{p}) & \leq {C∥Q\left(u,p\right)-p∥}_{0}{∥e_{p}-\Pi_{\mu }e_{p}∥}_{0}\\ \; & \leq C\mu^{\min\{1,\gamma \}}{(∥u∥}_{1+\gamma }+{∥p∥}_{\gamma })∥e_{p}-\Pi_{\mu }e_{p}{∥}_{0}. \end{array} \end{array}$$

Thus, from (5), (9), (23), (25) and Lemma 1, the right-hand side of (A1) can be estimated as

(A5) $$\begin{array}{c} \begin{array}{c} r.h.s\leq \hat{N}\left({∥\left(u,b\right)∥}_{1}+{∥\left(u_{\mu },b_{\mu }\right)∥}_{1}\right){∥\left(u-R\left(u,p\right),b-\Lambda b\right)∥}_{1}{∥\left(e_{u},e_{b}\right)∥}_{1}\\ \;\;+\sigma \mu \left({∥\left(u,b\right)∥}_{1}+{∥\left(u-R\left(u,p\right),b-\Lambda b\right)∥}_{1}\right){∥\left(e_{u},e_{b}\right)∥}_{1}\\ \;\;+C\mu^{\min\{1,\gamma \}}{(∥u∥}_{1+\gamma }+{∥p∥}_{\gamma })∥e_{p}-\Pi_{\mu }e_{p}{∥}_{0}\\ \;\leq \hat{N}\left(\frac{{∥F∥}_{*}}{\underline{v}}+\frac{{∥F∥}_{*}}{C_{min}}\right){∥\left(u-R\left(u,p\right),b-\Lambda b\right)∥}_{1}{∥\left(e_{u},e_{b}\right)∥}_{1}\\ \;\;+\sigma \mu \left(\frac{{∥F∥}_{*}}{\underline{v}}+{∥\left(u-R\left(u,p\right),b-\Lambda b\right)∥}_{1}\right){∥\left(e_{u},e_{b}\right)∥}_{1}\\ \;\;+C\mu^{\min\{1,\gamma \}}{(∥u∥}_{1+\gamma }+{∥p∥}_{\gamma })∥e_{p}-\Pi_{\mu }e_{p}{∥}_{0}\\ \;\leq C(\mu^{\min\{1,\gamma ,\tau \}}\left({∥u∥}_{1+\gamma }+{∥p∥}_{\gamma }+{∥b∥}_{\tau }+{∥\mathrm{curl}b∥}_{\tau }+{∥r∥}_{\tau +1}\right)\\ \;\;+\sigma {\underline{v}}^{-1}\mu {∥F∥}_{*}){\left(∥\left(e_{u},e_{b}\right){∥}_{1}^{2}+{∥e_{p}-\Pi_{\mu }e_{p}∥}_{0}^{2}\right)}^{\frac{1}{2}}. \end{array} \end{array}$$

It follows from (A2)–(A5) that

(A6) $$\begin{array}{c} \begin{array}{c} ∥\left(e_{u},e_{b}\right){∥}_{1}\leq C\mu^{\min\{1,\gamma ,\tau \}}{(∥u∥}_{1+\gamma }+{∥p∥}_{\gamma }+{∥b∥}_{\tau }\\ \;+{∥\mathrm{curl}b∥}_{\tau }+{∥r∥}_{\tau +1})+C\sigma {\underline{v}}^{-1}\mu {∥F∥}_{*}. \end{array} \end{array}$$

Using triangle inequality, (23), (25) and (A6) to obtain (28). From (2) and (17), we have

(A7) $$\begin{array}{c} a_{s}\left(u-u_{\mu },v\right)-b_{s}\left(p-p_{\mu },v\right)+b_{s}\left(q,u-u_{\mu }\right)+G\left(p-p_{\mu },q\right)\\ \;=c_{0}\left(u_{\mu }-u,u,v\right)+c_{0}\left(u_{\mu },\;u_{\mu }-u,v\right)+c_{1}\left(b-b_{\mu },v,b\right)\\ \;+c_{1}\left(b_{\mu },v,b-b_{\mu }\right)+\frac{\sigma \mu }{R_{e}^{-1}}a_{s}\left(u_{\mu },v\right)+G\left(p,q\right). \end{array}$$

Using (16), (5), (9), (14) and Lemma 1 to derive

(A8) $$\begin{array}{cc} \hat{\beta } & \left(R_{e}^{-1}∥\nabla \left(u-u_{\mu }\right){∥}_{0}+{∥p-p_{\mu }∥}_{0}\right)\\ \; & \leq \hat{N}\left({∥\left(u,b\right)∥}_{1}+{∥\left(u_{\mu },b_{\mu }\right)∥}_{1}\right){∥\left(u-u_{\mu },b-b_{\mu }\right)∥}_{1}\\ \; & \;+C_{max}\sigma \mu ∥\left(u_{\mu },b_{\mu }\right){∥}_{1}+C{∥p-\Pi_{\mu }p∥}_{0}\\ \; & \leq \hat{N}\left(\frac{{∥F∥}_{*}}{\underline{v}}+\frac{{∥F∥}_{*}}{C_{min}}\right){∥\left(u-u_{\mu },b-b_{\mu }\right)∥}_{1}\\ \; & \;+\sigma \mu \frac{C_{max}}{C_{min}}{∥F∥}_{*}+c_{2}\mu^{\min\{1,\gamma \}}{∥p∥}_{\gamma }\\ \; & \leq 2\underline{v}\sigma_{0}∥\left(u-u_{\mu },b-b_{\mu }\right){∥}_{1}+{C\sigma \mu ∥F∥}_{*}+C\mu^{\min\{1,\gamma \}}{∥p∥}_{\gamma }. \end{array}$$

From (3) and (17), and taking s=0 to get

(A9) $$\begin{array}{c} b_{m}\left(r-r_{\mu },c\right)=a_{m}\left(b-b_{\mu },c\right)+c_{1}\left(b-b_{\mu },u,c\right)+c_{1}\left(b_{\mu },u-u_{\mu },c\right). \end{array}$$

Applying (10), (5), (9) and Lemma 1, we have

(A10) $$\begin{array}{cc} \hat{\beta }{∥\nabla \left(r-r_{\mu }\right)∥}_{0} & \leq C_{max}∥\left(u-u_{\mu },b-b_{\mu }\right){∥}_{1}+\hat{N}∥\left(u-u_{\mu },b-b_{\mu }\right){∥}_{1}{∥\left(u,b\right)∥}_{1}\\ \; & \;+\hat{N}∥\left(u_{\mu },b_{\mu }\right){∥}_{1}{∥\left(u-u_{\mu },b-b_{\mu }\right)∥}_{1}\\ \; & \leq C_{max}∥\left(u-u_{\mu },b-b_{\mu }\right){∥}_{1}+\underline{v}\sigma_{0}{∥\left(u-u_{\mu },b-b_{\mu }\right)∥}_{1}\\ \; & \;+C_{min}\sigma_{1}{∥\left(u-u_{\mu },b-b_{\mu }\right)∥}_{1}. \end{array}$$

Combining (28) and (A8) with (A10), we can obtain (29). Further, to obtain (30), we need to introduce the dual problem [11]: For all (v,c,q,l)∈X×W×Q×S, find (w,Φ,s,t)∈X×W×Q×S such that

(A11) $$\begin{array}{c} A\left(v,c;w,\Phi \right)+C\left(u_{\mu },b_{\mu };v,c;w,\Phi \right)+C\left(v,c;u,b;w,\Phi \right)-B\left(s,t;v,c\right)\\ \;\;+B\left(q,l;w,\Phi \right)=\left(u-u_{\mu },b-b_{\mu };v,c\right). \end{array}$$

Taking (v,c;q,l)=(w,Φ;s,t) in (A11) and using (9), then

(A12) $$\begin{array}{c} C_{min}\left(1-\sigma_{1}\right)∥\left(w,\Phi \right){∥}_{1}\leq {∥\left(u-u_{\mu },b-b_{\mu }\right)∥}_{0}. \end{array}$$

Assuming that the region Ω is sufficiently smooth (see Theorem 2 of [11]), then the following regularity error estimates are available

$$\begin{array}{c} \begin{array}{c} {∥\left(w,\Phi \right)∥}_{2}+{∥\left(s,t\right)∥}_{1}\\ \;\leq C∥\left(u-u_{\mu },b-b_{\mu }\right){∥}_{0}\\ \;\;+\hat{N}\left({∥\left(u,b\right)∥}_{1}+{∥\left(u_{\mu },b_{\mu }\right)∥}_{1}\right){∥\left(w,\Phi \right)∥}_{1}^{\frac{1}{2}}{∥\left(w,\Phi \right)∥}_{2}^{\frac{1}{2}}\\ \;\leq C∥\left(u-u_{\mu },b-b_{\mu }\right){∥}_{0}+2C_{min}\sigma_{1}∥\left(w,\Phi \right){∥}_{1}^{\frac{1}{2}}{∥\left(w,\Phi \right)∥}_{2}^{\frac{1}{2}}\\ \;\leq C∥\left(u-u_{\mu },b-b_{\mu }\right){∥}_{0}+C\sigma_{1}^{2}∥\left(w,\Phi \right){∥}_{1}+\frac{1}{2}{∥\left(w,\Phi \right)∥}_{2}\\ \;\leq C∥\left(u-u_{\mu },b-b_{\mu }\right){∥}_{0}+\frac{1}{2}{∥\left(w,\Phi \right)∥}_{2}, \end{array} \end{array}$$

thereby

(A13) $$\begin{array}{c} {∥\left(w,\Phi \right)∥}_{2}+{∥\left(s,t\right)∥}_{1}\leq C{∥\left(u-u_{\mu },b-b_{\mu }\right)∥}_{0}. \end{array}$$

Finding $v=u-u_{\mu },c=b-b_{\mu },q=l=0$ such that

(A14) $$\begin{array}{c} A\left(u-u_{\mu },b-b_{\mu };w,\Phi \right)+C\left(u_{\mu },b_{\mu };u-u_{\mu },b-b_{\mu };w,\Phi \right)\\ \;+C\left(u-u_{\mu },b-b_{\mu };u,b;w,\Phi \right)-B\left(s,t;u-u_{\mu },b-b_{\mu }\right)\\ =(u-u_{\mu },b-b_{\mu };u-u_{\mu },b-b_{\mu }). \end{array}$$

Subtracting (2) and (3) from (18), and taking q=l=0, for all $\left(v,c\right)\in X_{\mu }\times W_{\mu }$, we have

(A15) $$\begin{array}{c} A\left(u-u_{\mu },b-b_{\mu };v,c\right)+C\left(u_{\mu },b_{\mu };u-u_{\mu },b-b_{\mu };v,c\right)\\ \;+C\left(u-u_{\mu },b-b_{\mu };u,b;v,c\right)-B\left(p-p_{\mu },r-r_{\mu };v,c\right)\\ \;-\frac{\sigma \mu }{R_{e}^{-1}}a_{s}\left(u_{\mu },v\right)=0. \end{array}$$

Subtract (A15) from (A14) to get

(A16) $$\begin{array}{c} A(u-u_{\mu },b-b_{\mu };w-v,\Phi -c)\\ \;+C(u_{\mu },b_{\mu };u-u_{\mu },b-b_{\mu };w-v,\Phi -c)\\ \;+C(u-u_{\mu },b-b_{\mu };u,b;w-v,\Phi -c)\\ \;+B(q_{\mu }^{\prime }-s,l_{\mu }^{\prime }-t;u-u_{\mu },b-b_{\mu })\\ \;-B(p-p_{\mu },r-r_{\mu };w-v,\Phi -c)\\ \;+\frac{\sigma \mu }{R_{e}^{-1}}a_{s}\left(u_{\mu },v\right)={∥\left(u-u_{\mu },b-b_{\mu }\right)∥}_{0}^{2}, \end{array}$$

where $q_{\mu }^{\prime }\in Q_{\mu }$ and $l_{\mu }^{\prime }\in S_{\mu }$ are the approximation of *s* and *t*, respectively. Similar to the derivation in the literature [41] (see the proof of Theorem 3.2), we get

$${∥\left(w,\Phi \right)∥}_{1}\leq C\mu {∥\left(w,\Phi \right)∥}_{2},\;w\in H^{2}\left(\Omega \right),\;\Phi \in H^{2}\left(\Omega \right).$$

Taking v=R(w,s),c=ΛΦ in (A16), Applying (5), (7), (9), (23), (25), (A13) to get

$$\begin{array}{cc} \; & ∥\left(u-u_{\mu },b-b_{\mu }\right){∥}_{0}^{2}\\ \; & \;\leq \bar{v}∥\left(u-u_{\mu },b-b_{\mu }\right){∥}_{1}{∥\left(w-R\left(w,s\right),\Phi -\Lambda \Phi \right)∥}_{1}\\ \; & \;\;+\hat{N}{(∥\left(u,b\right)∥}_{1}∥\left(u-u_{\mu },b-b_{\mu }\right){∥}_{1}{∥\left(w-R\left(w,s\right),\Phi -\Lambda \Phi \right)∥}_{1}\\ \; & \;\;+\hat{N}∥\left(u_{\mu },b_{\mu }\right){∥}_{1}∥\left(u-u_{\mu },b-b_{\mu }\right){∥}_{1}{∥\left(w-R\left(w,s\right),\Phi -\Lambda \Phi \right)∥}_{1}\\ \; & \;\;+∥\left(u-u_{\mu },b-b_{\mu }\right){∥}_{1}∥\left(s-q_{\mu }^{\prime },t-l_{\mu }^{\prime }\right)∥\\ \; & \;\;+{∥\left(w-R\left(w,s\right),\Phi -\Lambda \Phi \right)∥}_{1}∥\left(p-p_{\mu },r-r_{\mu }\right)∥\\ \; & \;\;+\sigma \mu ∥\left(u_{\mu },b_{\mu }\right){∥}_{1}(∥\left(w-R\left(w,s\right),\Phi -\Lambda \Phi \right){∥}_{1}+∥\left(w,\Phi \right){∥}_{1})\\ \; & \;\leq C\mu ∥\left(u-u_{\mu },b-b_{\mu }\right){∥}_{1}{∥\left(u-u_{\mu },b-b_{\mu }\right)∥}_{0}\\ & \;\;+C\mu ∥\left(p-p_{\mu },r-r_{\mu }\right)∥∥\left(u-u_{\mu },b-b_{\mu }\right){∥}_{0}+C\mu^{2}{∥\left(u-u_{\mu },b-b_{\mu }\right)∥}_{0}, \end{array}$$

which together with (28) and (29) yield the desired result (30). The proof ends.   □
